# Successful Non-fusion Management of a Healed Pediatric Odontoid Synchondrosis Fracture: A Case Report

**DOI:** 10.7759/cureus.71108

**Published:** 2024-10-08

**Authors:** Adam Abu-Gameh, Hezi Ferster, Dimitry Sheinis, Nissim Ohana

**Affiliations:** 1 Orthopaedics, Soroka Medical Center, Beer Sheva, ISR; 2 Orthopaedics, Soroka Medical Center, Beer sheva, ISR; 3 Orthopaedic Surgery, Meir Medical Center, Kfar Sabba, ISR

**Keywords:** case report, cervical spine mobility, non-fusion treatment, odontoid synchondrosis fracture, pediatric spine injury

## Abstract

Odontoid synchondrosis fractures are rare pediatric spinal injuries that pose significant diagnostic and treatment challenges. These fractures, typically occurring in the C-2 vertebra, often result from high-energy trauma and are difficult to diagnose due to subtle radiographic findings. We present the case of a 4-year-old male who sustained a head injury while playing on a trampoline. Initially diagnosed with a head injury and discharged without noting any neck complaints, the patient returned to the ER two months later with neck pain and stiffness. Radiographs revealed a C2 fracture at the base of the odontoid process, confirmed by CT and MRI scans. The patient underwent open reduction and HALO fixation to manage the displaced sub-dental synchondrosis fracture. The patient was admitted to the operating room for reduction and immobilization under general anesthesia. After an unsuccessful closed reduction attempt, an open reduction was performed followed by the application of a HALO vest. Postoperative care included follow-up appointments at 6 weeks, 12 weeks, and 16 months, which showed good alignment, significant bone union, and complete fracture healing. At the most recent check-up, six years post-injury, the patient demonstrated full neck mobility without pain or neurological symptoms. This case highlights the successful management of a pediatric odontoid synchondrosis fracture with non-fusion treatment. Even with delayed intervention, appropriate and timely treatment led to excellent outcomes, ensuring proper healing and preserving the functional mobility of the cervical spine. This underscores the importance of thorough initial assessment and the potential for successful non-fusion treatment strategies in pediatric cervical spine injuries.

## Introduction

Odontoid synchondrosis fractures are rare pediatric spinal traumas that are challenging to diagnose due to the unique anatomical and biomechanical characteristics of children's upper cervical spine [1-3]. In 1952, Bailey [4] identified four primary ossification centers in the axis present at birth: one for the odontoid, one for the body, and two for the neural arches. The cartilage lines between these centers resemble a synchondrosis rather than a typical epiphyseal plate, remaining cartilaginous and potentially weak until they fuse between the ages of 3 and 6 years. High-energy trauma or significant head injuries in children within this age group can lead to these fractures [5,6]. However, there are cases where only minor trauma or no prior injury has been reported, including one instance of this injury occurring at birth [7].

Diagnosing odontoid synchondrosis fractures can be difficult due to the subtlety of radiographic findings and the non-verbal nature of young patients. Advanced imaging techniques such as CT and MRI are often required for accurate diagnosis, as plain radiographs may initially appear normal or show minimal displacement [8,9]. MRI is particularly useful in assessing soft tissue damage and spinal cord involvement, providing crucial information on ligamentous injuries that may accompany the fracture.

Due to its rarity [1-3, 5-6], treatment options for odontoid synchondrosis fractures are mainly documented in case reports and small clinical series. Traditionally, conservative treatment was common, even in delayed diagnoses [6,10-12]. However, recent trends favor surgical intervention, usually involving posterior C1-C2 arthrodesis [1,2,13,14]. Unfortunately, fusing the second cervical vertebra to an adjacent one can limit future neck rotation. Conservative treatment with a halo vest carries risks such as pin loosening, infection, and discomfort.

We report a case of a child diagnosed 8 weeks late with a synchondrosis fracture of the axis. The fracture was treated operatively with open reduction, followed by external stabilization using a HALO orthosis, without internal fixation.

## Case presentation

**Figure 1:**
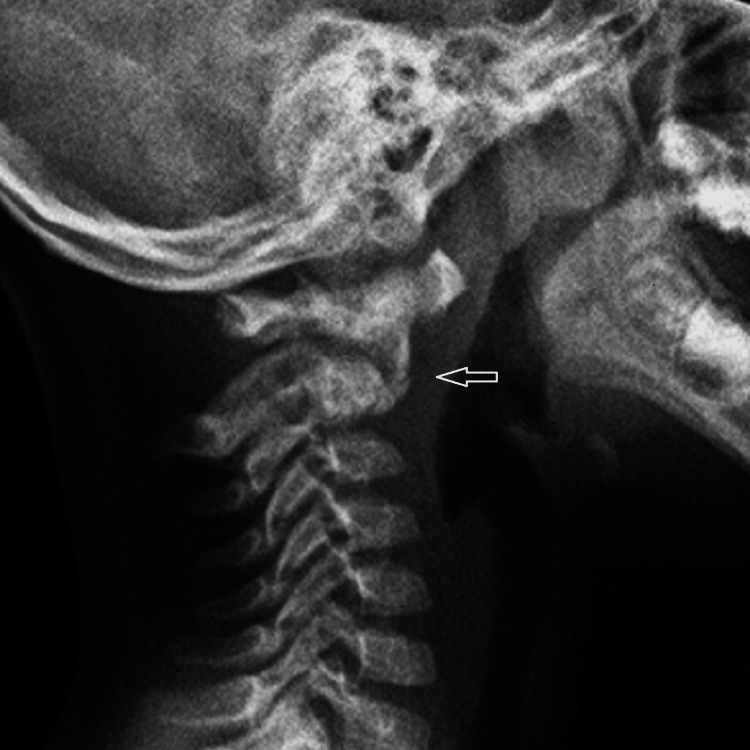
Plain lateral cervical spine radiograph A plain lateral radiograph, taken two months following injury, shows an angulated fracture at the base of the odontoid process (C2) with displacement. An arrow points to the fracture line and the displacement of the dens.

A healthy 35 kg, 4-year-old boy sustained a head injury on a home trampoline, briefly losing consciousness. He was stable and neurologically intact upon arrival at the ER, where a head CT was performed, and he was discharged without neck complaints. Two months later, his parents noticed his inability to extend or rotate his head, leading to a return to the ER with mild neck pain and stiffness. Initially overlooked, his symptoms worsened, revealing significant neck stiffness and reduced range of motion, but no neurological deficits. A lateral view plain radiograph is shown in Figure 1.

**Figure 2:**
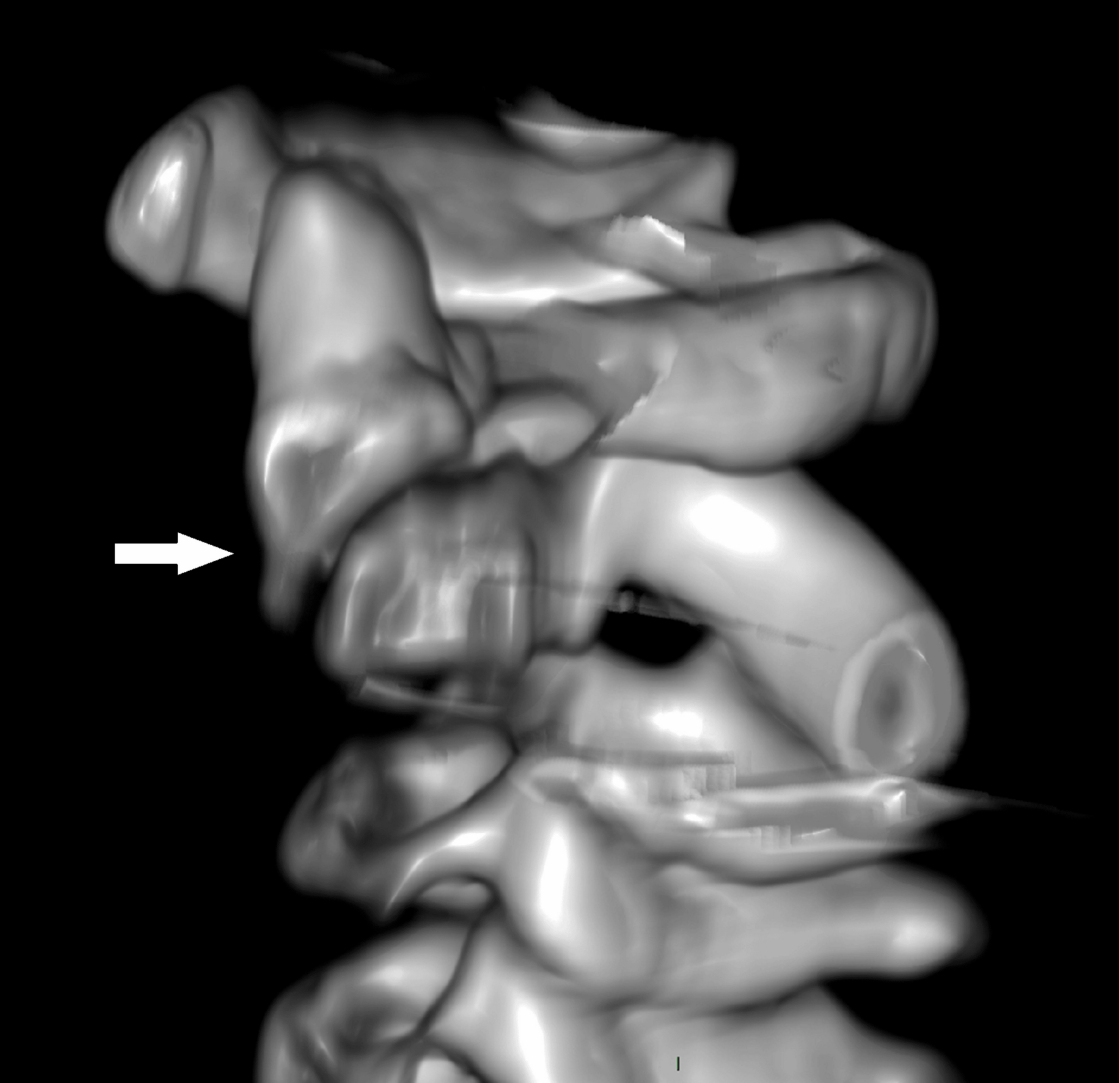
Sagittal reconstructed 3D CT scan Sagittal reconstructed CT scan demonstrating a displaced sub-dental synchondrosis fracture with signs of delayed healing. An arrow points to the fracture and highlights the displacement of the dens.

CT scan revealed a displaced sub-dental synchondrosis fracture with no clear signs of healing (Figure 2).

An MRI was ordered to rule out spinal cord involvement but showed soft tissue swelling around the fracture site.

Treatment

**Figure 3:**
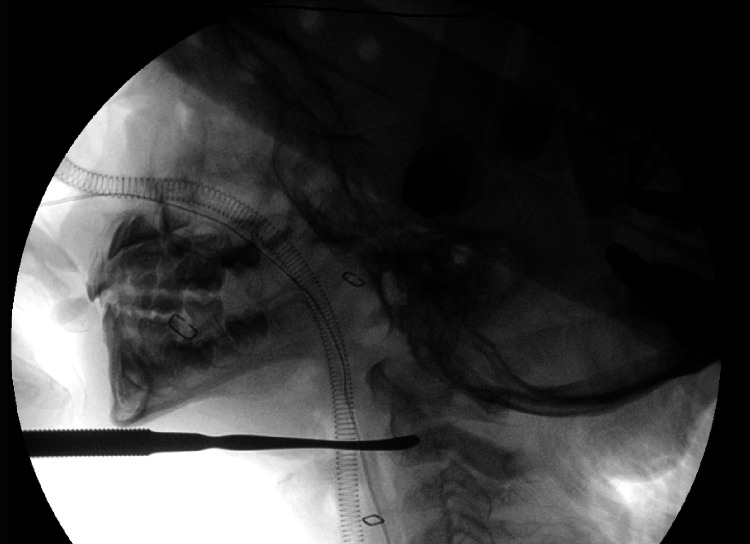
Intraoperative lateral c-spine fluoroscopy The image shows the use of a freer elevator for osteoclasis by inserting it between the dens and the C2 body.

The patient underwent reduction and immobilization under general anesthesia. Nasal intubation was used, and a HALO crown was attached to a traction device, gradually increasing to 6 kg. Despite multiple attempts at trans-oral closed manipulation, it failed, suggesting a healed fracture, leading to the decision for open reduction. A right cervical, submandibular incision exposed the deformed C2 anteriorly. An osteoclasis was performed at the fracture site (Figure 3), and the dislocated part was successfully restored to its anatomical position under X-ray fluoroscopy.

Following reduction, a HALO vest was applied for alignment and immobilization. The crown screws were re-tightened the next morning. The patient was allowed to mobilize independently and was discharged after 24 hours of observation.

Follow-up

**Figure 4:**
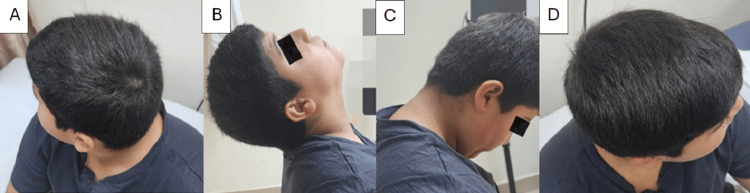
Clinical photograph of the patient's head movement The image is showing the patient’s full range of neck motion, with normal right rotation (A), extension (B), flexion (C) and left rotation (D), six years post-treatment.

**Figure 5:**
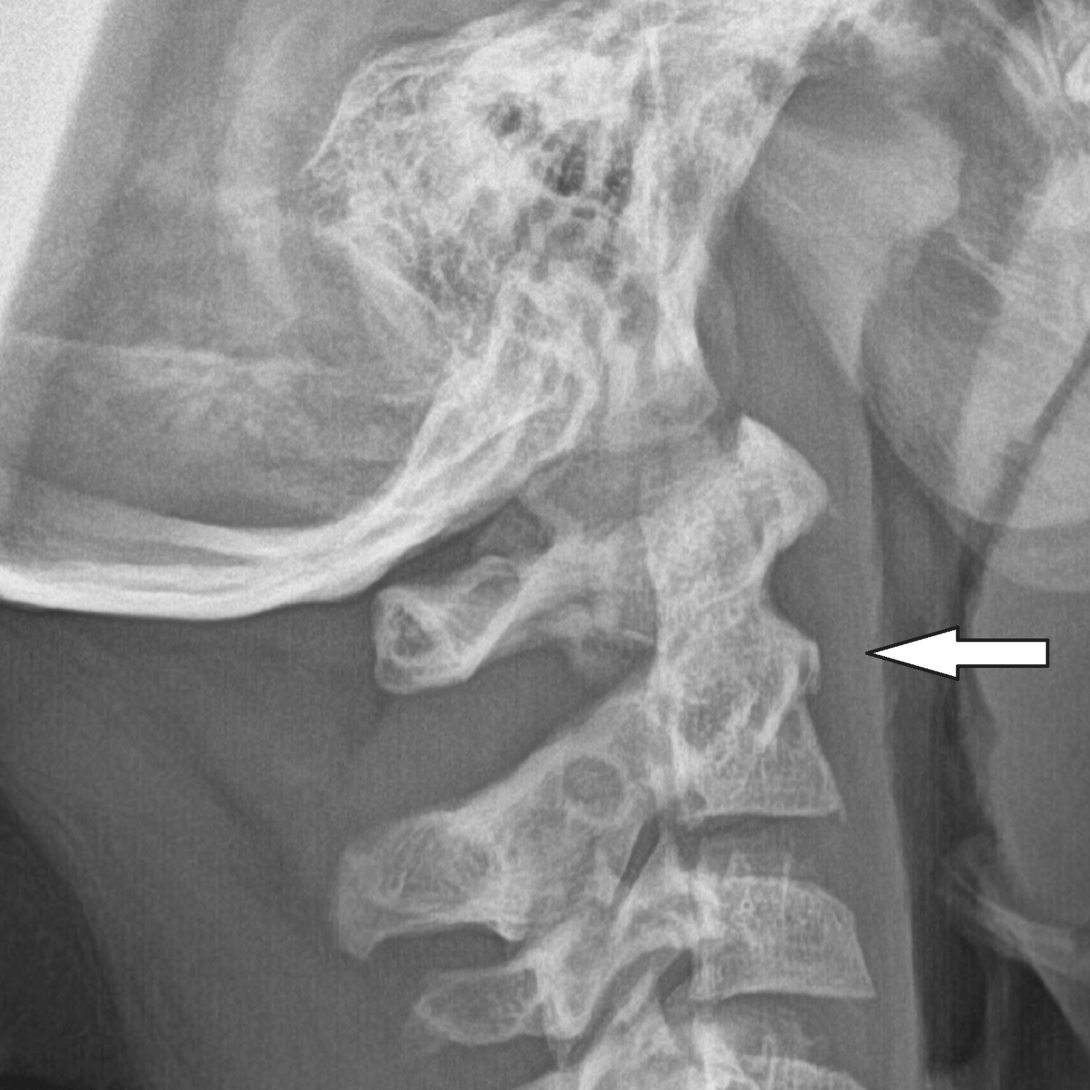
Plain lateral C-spine radiographic Plain lateral radiographic assessment, six years following surgery, confirming complete healing and remodeling of the fracture. An arrow points to the normal shape and size of the C2 vertebra.

Follow-up ensured proper healing and halo adjustments. At 6 weeks, radiographs showed good alignment and early healing. By 8 weeks, stable alignment allowed for halo removal and a rigid collar for 4 weeks. At 16 months, full healing and remodeling were confirmed, with complete neck mobility and no symptoms. At the six-year follow-up, the patient had normal neck function (Figure 4), and radiographs showed a normal second cervical vertebra with no growth issues (Figure 5).

## Discussion

Odontoid synchondrosis fractures predominantly affect young children under the age of seven, with a mean age of approximately 2.8 years, as reported in various studies [1-3,5,6]. Our patient, who was 4 years old, falls within this age range. The incidence of these fractures is higher in this age group due to the biomechanical vulnerability of the synchondrosis and the relatively large size of children's heads, which can act as a pendulum during high-speed trauma [1-9]. The trauma our patient experienced-a head injury while jumping on a trampoline-fits this described mechanism of hyperflexion or high-energy trauma, similar to motor vehicle accidents or falls from significant heights. Anatomical predisposition, coupled with underdeveloped neck muscles and horizontally oriented facet joints, increases the risk of odontoid fractures in this age group [12,15]. The cartilaginous synchondrosis at the base of the dens is prone to shear forces, leading to fractures with varying displacement and angulation [16]. Most cases are diagnosed early due to significant injury history, but delayed diagnoses, like in our case, often follow minor trauma [10,16]. The rarity of this injury and low awareness among ER pediatricians likely contributed to the delay.

The clinical presentation of odontoid synchondrosis fractures can range from mild neck pain to severe neurological deficits [16], depending on the extent of the injury. In many cases, children may present with torticollis, limited neck mobility, or signs of spinal cord injury.

Diagnosing these fractures is challenging due to subtle radiographic findings and the non-verbal nature of young patients. Plain radiographs may appear normal or show minimal displacement, often requiring advanced imaging like CT, which can also be inconclusive in non-displaced fractures16. MRI is essential for a thorough evaluation.

Radiologic classification of odontoid synchondrosis fractures

Recognizing common injury patterns and normal developmental anatomy during radiologic assessment is crucial [17]. Hosalkar et al. [15] introduced a simple classification system based on the degree of anterior displacement of the odontoid fracture in children with open basilar synchondrosis, classifying our patient as Subtype b (10-100% anterior displacement). Rusin et al. [17,18] published a more detailed classification for C2 synchondrosal fractures based on specific synchondroses and displacement, though its complexity may limit routine clinical use.

This case underscores the challenges of managing neglected fused sub-dental synchondrosis. Unlike non-displaced or reducible fractures treatable conservatively [7,10,11,16], our case involved a freshly fused, angulated fracture. Early intervention usually involves conservative measures with a halo-jacket, which carries risks, including a 43.3% crown complication rate and an 11.4% non-union rate [19]. Delayed cases may require more invasive surgery.

Most published cases suggest C1-C2 arthrodesis, though this surgery has been offered in only a minority of cases and is mainly reported in more recent years [1,19]. Our case shows that non-fusion techniques can achieve stable fracture healing while preserving motion, crucial for children with a lifetime ahead under our treatment recommendations.

We weighed the risks of malunion and potential bone growth disorders against the benefits of preserving motion. Opting for a non-fusion approach, with C1-C2 fusion as a backup, the parents consented. The fracture healed well, with no developmental issues, axis changes, or loss of neck mobility.

## Conclusions

Pediatric head and neck trauma requires careful evaluation for cervical spine injuries, as subtle presentations can lead to delayed diagnoses. This case demonstrates the successful management of a pediatric odontoid synchondrosis fracture using non-fusion treatment, showing that even with delayed intervention, favorable outcomes are possible. The patient retained full cervical mobility without long-term complications following open reduction and halo fixation. This highlights the effectiveness of non-fusion treatment strategies in preserving both the structural integrity and functionality of the cervical spine. Early and accurate diagnosis is crucial, but this case provides evidence that non-fusion approaches can still achieve excellent results even with delayed treatment.
